# Investigation of Liquid Oils Obtained by Thermo-Catalytic Degradation of Plastic Wastes in Energy Recovery

**DOI:** 10.3390/molecules30091959

**Published:** 2025-04-28

**Authors:** Mihaela Vlassa, Miuța Filip, Simion Beldean-Galea, Didier Thiébaut, Jerôme Vial, Ioan Petean

**Affiliations:** 1 Raluca Ripan Institute for Research in Chemistry, Babeş-Bolyai University, 400294 Cluj-Napoca, Romania; mihaela.vlassa@ubbcluj.ro; 2 Faculty of Environmental Science and Engineering, Babeș-Bolyai University, 400294 Cluj-Napoca, Romania; simion.beldean@ubbcluj.ro; 3 Chemistry, Biology and Innovation Department, École Supérieure de Physique et de Chimie Industrielles, 75005 Paris, France; didier.thiebaut@espci.psl.eu (D.T.); jerome.vial@espci.psl.eu (J.V.); 4 Faculty of Chemistry and Chemical Engineering, Babeș-Bolyai University, 400028 Cluj-Napoca, Romania; ioan.petean@ubbcluj.ro

**Keywords:** plastic wastes, catalytic degradation, liquid oils, GC × GC-qMS

## Abstract

The most efficient technique for resolving the issue of plastic waste disposal is by converting the wastes into high-quality liquid oils through thermal and catalytic pyrolysis. The objective of this work was to study the composition of liquid oils obtained by thermal and catalytic degradation of plastic wastes containing polystyrene (PS), polyvinyl chloride (PVC), and polyethylene terephthalate (PET). The clay catalysts were characterized by N_2_ adsorption–desorption isotherms (BET), Scanning Electron Microscopy (SEM) and Fourier transform Infrared Spectrometry (FTIR), Polarized Optical Microscopy (POM), Atomic Force Microscopy (AFM). The effect of temperature and clay catalyst type on the yields of the end-products resulting in thermo-catalytic degradation of PS has been evaluated. Degradation of PS showed the highest liquid oil production at 86.85% in comparison to other plastic types. The characterization of the liquid oils was performed by comprehensive two-dimensional gas chromatography coupled with single quadrupole mass spectrometry (GC × GC-qMS). In liquid oils of PS, eighteen principal compounds (of groups: linear hydrocarbons, mono-aromatics, and di-aromatics) were identified. In the liquid oils of the plastic waste mixture, twenty-four principal compounds (of groups: linear hydrocarbons, mono-aromatics, oxygen-containing aromatic, di-aromatics, and tri-aromatics) were identified. The liquid oils were investigated in order to reconvert them as styrene monomers or other chemicals in energy recovery.

## 1. Introduction

Plastic pollution has been labeled as a wicked problem and a crisis due to the levels of plastic use and associated wastes, which have increased exponentially. Investments in circular technologies like feedstock recycling or existing techniques with environmental viability could be the key to solving this problem. In the past few years, pyrolysis has attracted much attention in the industrial and scientific communities as a promising and versatile platform to convert plastic wastes into valuable resources [1,2].

Thermal and catalytic cracking technologies used in recycling plastic wastes are important, eco-friendly, and widely applicable. The end products of thermal or catalytic degradation of plastic wastes can serve as an additional source of chemicals and energy. Plastics wastes are part of the valuable carbon resources, and the most used plastics are polyethylene, including high-density polyethylene, low-density polyethylene, polypropylene, polystyrene (PS), polyethylene terephthalate (PET), and polyvinyl chloride (PCV) [1].

The pyrolysis of plastic solid wastes has gained importance due to its having better advantages in dealing with environmental pollution and the reduction of the carbon footprint of plastic products. Through pyrolysis, the emissions of carbon monoxide and carbon dioxide, compared to in combustion and gasification, were minimized; thus, the pyrolysis oils have attracted a lot of interest as energy resources and chemicals [2]. For selective removal of unsaturated hydrocarbons and HCl released from the pyrolysis of polyvinyl chloride catalytic sorbent, containing 5% NiO loaded on CaCO_3_ support (Ni-Ca) and pristine CaCO_3_ (Ca), was used [2].

The pyrolysis oil is a mixture of paraffins, olefins, naphthenes, aromatics, and sulfur-, nitrogen-, oxygen-containing compounds and their composition depends on the type of plastic wastes pyrolyzed [3]. Various researchers studied the conversion of plastic wastes into useful fuel products by using the thermal and catalytic process [2,4,5,6,7]. The conversion of polystyrene into its monomers is also reported [5,8,9].

Catalytic pyrolysis has an advantage over conventional thermal pyrolysis processes in the recovery of fuel from plastic wastes [10]. Various heterogeneous catalysts have been used, but the most commonly used are zeolites (HY, ZSM-5) [11], mesoporous aluminosilicates (Al-MCM-41 and Al-SBA-15), or metal oxides, such as MgO, Al_2_O_3_, and ZnO, which are more suitable for the production of polar and aromatic products [1,6,8,10].

Also, synthesized waste cotton fabric-activated carbon samples (WCF-AC) were utilized as a solid acid catalyst in the catalytic pyrolysis of low-density polyethylene for conversion into fuels [12]. Carbon and activated carbon, synthesized from different biomass, such as rice husk, corn husk, and coconut sheath, were used in microwave pyrolysis of PS and polypropylene waste, with styrene recovery in product oil at 67.58% [13].

Natural clay catalysts, including nanoclay, montmorillonite, kaolin, and hydrotalcite, were used as catalysts for the pyrolysis of mixed plastic (PET, PS, polypropylene, low-density polyethylene, and high-density polyethylene) for fuel and energy recovery. Clay catalysts improved oil yields and the proportion of gasoline or diesel fuels in catalytic pyrolysis of mixed plastic wastes [5,14]. The phyllosilicate-derived heterogeneous catalysts (acid-activated clays, ion-exchanged clays, and layered double hydroxides) exhibit excellent catalytic activity for biodiesel production [15].

The catalytic pyrolysis of polystyrene (PS) for the purpose of obtaining a styrene-rich liquid shows that the resulting pyro-oil yield was between 89 and 96 wt.%, depending on the catalyst used (ilmenite, olivine, calcium oxide, or dolomite). The main composition of oil was styrene monomer, styrene dimer, and styrene trimer, as well as other light aromatic compounds [15,16]. A high yield of aromatic hydrocarbons was also obtained in the catalytic fast pyrolysis of PS with HZSM-5, Hβ, HY, and ultra-stable Y [1,10].

Determination of the composition of the plastic pyrolysis oil is usually performed by conventional one-dimensional gas chromatography coupled with mass spectrometry (GC-MS) [5,16,17]. However, GC-MS has limited separation power when analyzing ultra-complex samples [16].

Comprehensive two-dimensional gas chromatography (GC × GC) coupled with mass spectrometry (MS) is a state-of-the-art technique that allows the separation power to be increased by exploiting two different separation mechanisms, thus improving fingerprinting sensitivity and effectiveness of sample classification [18,19,20,21].

The application of GC × GC for the characterization of petroleum products, plastic waste pyrolysis oils, and complex olefin mixtures has been described by various authors [19,22,23].

Products derived from the chemical recycling of plastic wastes comprise a complex mixture. In terms of both group type and carbon number, the following were found in the pyrolysis oil: hydrocarbon groups (n-paraffins, iso-paraffins, olefins and naphthenes, monoaromatics, naphtheno-aromatics, diaromatics, naphtheno-diaromatics, tri-aromatics, naphtheno tri-aromatics and tetra-aromatics), nitrogen (nitriles, pyridines, quinolines, indole, caprolactam, etc.), sulfur (thiols/sulfides, thiophenes/disulfides, benzothiophenes, dibenzothiophenes, etc.), and oxygen-containing compounds (ketones, phenols, aldehydes, ethers, etc.) [22,23,24].

In this work, the liquid oils obtained individually and in a mixture from the thermal degradation of some plastic wastes (PS, PVC, and PET) were investigated. The originality of this study consists of how Romanian natural clays were used as catalysts in the degradation of polystyrene waste, with the specification that catalysts are eco-friendly and non-toxic and have a low cost. Also, the influence of the temperature and the clay catalysts on the yield of the end-products obtained by thermal and catalytic degradation of PS was investigated. The clay catalysts were characterized by N_2_ adsorption–desorption isotherms (BET), Scanning electron microscopy (SEM), Fourier transform infrared spectrometry (FTIR), Polarized Optical Microscopy (POM), and Atomic Force Microscopy (AFM). For the characterization of liquid oils and better identification of chemicals, the GC × GC coupled with single quadrupole mass spectrometry (GC × GC-qMS) has been used. The GC × GC-qMS analyses provide a new insight into the composition of liquid oils obtained from the thermal degradation of waste plastics in order to help with the recovery of monomers, fuel, and chemicals.

## 2. Results

### 2.1. Characterization of Clay Catalysts

#### 2.1.1. Determination of Surface Area and Pore Distribution

**Figure 1:**
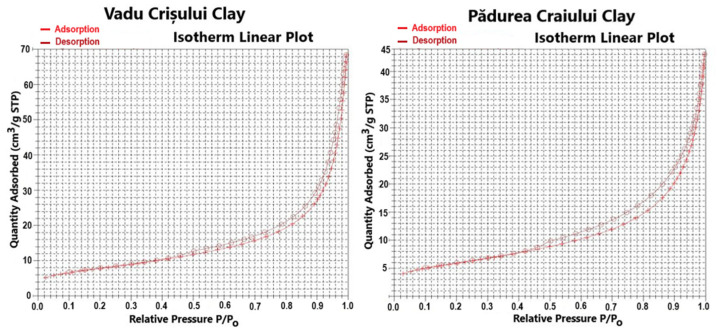
N_2_ adsorption–desorption isotherms at 77K of the studied catalysts.

The determination of surface area and pore distribution of catalysts is important for understanding dispersion for the active metals. A higher surface area of support results in higher dispersion of the active metals. Also, the pore size determines the accessibility of reactants to the active sites and the ability of diffusion of products back to the bulk fluid [25]. Therefore, the surface area and porosity characteristics of the studied clay catalyst are presented in Figure 1.

The nitrogen adsorption–desorption isotherms shown in Figure 1 are predominantly type I (Langmuir) combined with type IV isotherms. The smaller pores fill at lower relative pressures (p/p_0_, where p_0_ is the saturation pressure of the adsorptive), and larger pores fill at higher relative pressures. The relation between pore filling pressures and pore size is clear in the progression in isotherm shape and suggests that the catalysts are micro and mesoporous materials [25].

**Figure 2:**
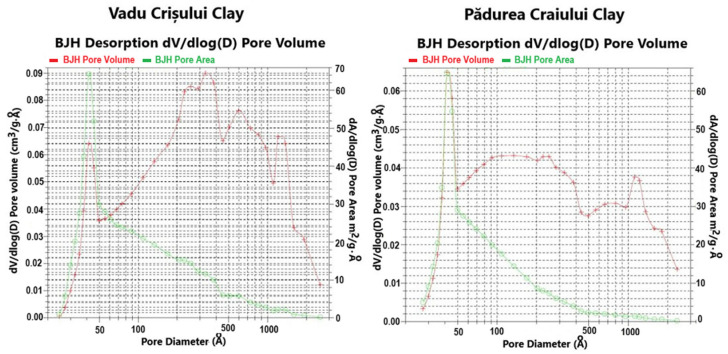
Pore size cumulative/derived distribution curve of the studied catalysts.

Table 1 and Figure 2 present the surface area and porosity characteristics and the pore size cumulative/derived distribution curves (pore volume, pore area) of the studied catalysts.

The Vadu Crişului clay catalyst has a greater value of BET surface area of 27.80 m^2^·g^−1^ than the BET surface area of Pădurea Craiului clay catalyst of 21.01 m^2^·g^−1^. The values of meso- and macropore volumes are 0.108 cm^3^·g^−1^ for the Vadu Crişului clay catalyst and 0.067 cm^3^·g^−1^ for the Pădurea Craiului clay catalyst. The distribution curve of pore size volume shows that the mesopores with diameters between 35 and 50 Å are predominant and occupy the largest volume [25].

#### 2.1.2. SEM-EDX Analysis

**Figure 3:**
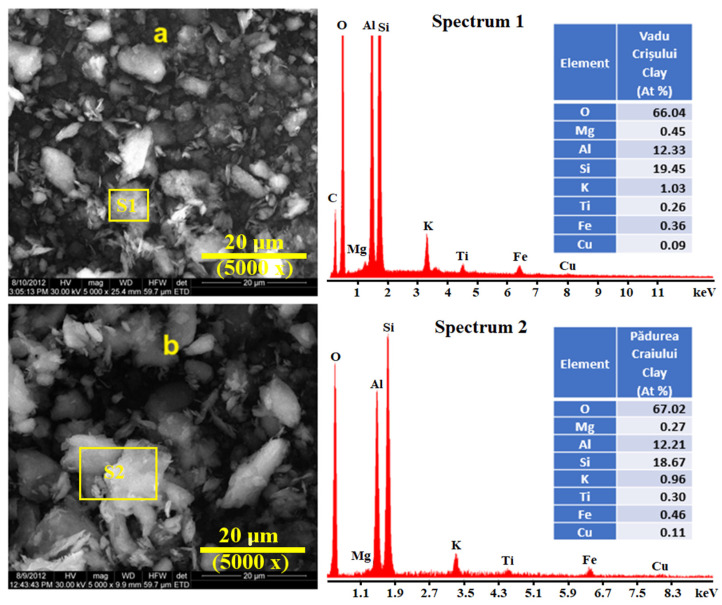
SEM microphotographs of the studied catalysts: (**a**) Vadu Crișului Clay; (**b**) Pădurea Craiului Clay. The EDX spectra and elemental composition of the studied catalysts are presented.

The morphology of the catalysts was determined by the SEM images, and the EDX spectra show the elemental composition (Figure 3).

The chemical composition of both investigated Romanian clay catalysts was found to be similar, with SiO_2_ and Al_2_O_3_ as the main components and K_2_O, MgO, TiO_2_, and Fe_2_O_3_ as minor components. These clay catalysts are the kaolinite type, and the Vadu Crisului clay catalyst was previously characterized [5].

Kaolinite is a 1:1 hydrous aluminosilicate with the generalized mineral formula Al_2_O_3_·2SiO_2_⋅2H_2_O that contains minerals composed of one silica tetrahedral layer associated with one alumina octahedral layer in an alternate fashion [15].

Figure 3a,b shows the structure of the studied clay catalysts, which are constituted by particles of different sizes and larger crystals. The morphology and different shapes are formed by agglomeration of the macropores. All the measures were carried out at 20 µm scale for better correlation. The microporous structure is not observed by SEM. These images reveal a small variation in morphology, the Vadu Crişului clay catalyst shows the formation of disordered structures, while the Pădurea Craiului clay catalyst shows larger particle aggregates with smooth surfaces. This confers to catalysts a highly heterogeneous internal structure.

#### 2.1.3. FTIR Analysis

**Figure 4:**
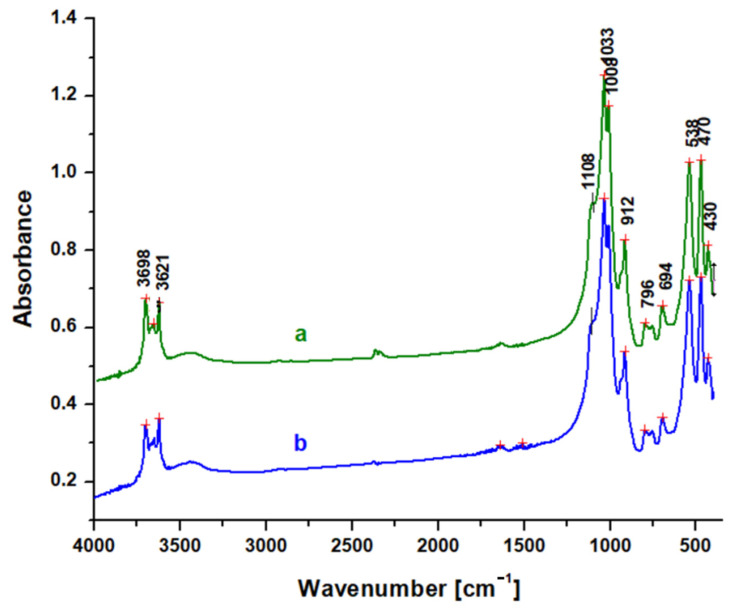
FTIR spectra of studied clay catalysts: (a) Vadu Crișului and (b) Pădurea Craiului.

In general, the constituent units of clay minerals include hydroxyl groups, tetrahedral silicate/aluminate anions, octahedral metal cations, and interlayer cations. The spectra of Figure 4 demonstrate well crystalline kaolinite [26].

FTIR spectra can be useful for obtaining structural information about catalysts, including the channel size and the cation substitution (Si^4+^ by Al^3+^) in the tetrahedral sites of zeolite minerals.

In FTIR spectra, OH-stretching modes lie in the spectral region of 3400–3750 cm^−1^. The bands at 3696 and 3620 cm^−1^ are characteristic of the kaolin group in general and arise from OH-stretching modes. Metal-O-H bending modes occur in the 600–950 cm^−1^ region. The OH deformation bands of kaolinite are situated at 913 cm^−1^ [26].

The Si-O vibration is evidenced by the very strong absorption bands from 1108, 1049, and 1008 cm^−1^, while the bands at 538 and 470 cm^−1^ appear due to Al-O-Si and Si-O-Si bending vibrations, respectively [27]. The weak band from 796 cm^−1^ and 694 cm^−1^ occurs due to the vibrations of internal oxygen bridges Si-O-Si, and it is a diagnosis for kaolinite [28].

#### 2.1.4. POM Analysis

Crossed Polarized light Optical Microscopy (POM) analysis has the benefit of identifying the mineral particles within a granular mixture like clay on their interference colors related to the birefringence.

**Figure 5:**
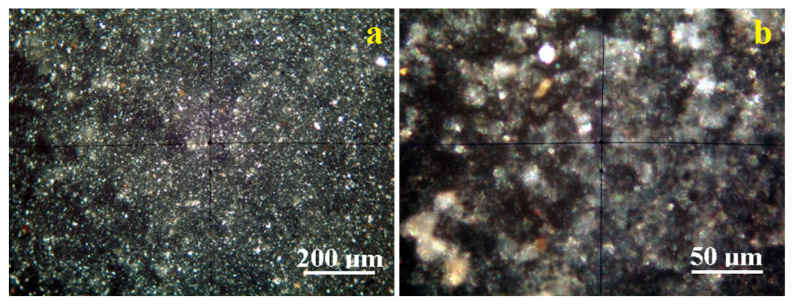
Mineralogical optical microscopy images observed in cross-polarized light for Vadu Crișului clay catalyst: (**a**) microstructural overview; (**b**) microstructural detail.

Figure 5a reveals the microstructural overview of the Vadu Crișului clay. It evidences a uniform spread of fine particles with a bright white color characteristic of kaolinite. They are surrounded by a pale bluish hue corresponding to the finest kaolinite particles, in good agreement with the literature data.

These finest kaolinite particles are better observed in the microstructural detail, Figure 5b. They have preponderantly tabular-lamellar habits and sizes of about 1–2.5 μm that tend to agglomerate in clusters of about 20–30 μm. The cluster’s color is bluish-white, and its intensity varies with the kaolinite particle disposal relative to the optical axis of the microscope. Fine traces of muscovite (brown–pink color) and quartz (green–gray color) are observed uniformly distributed among kaolinite particles having a size range of 1–5 µm.

**Figure 6:**
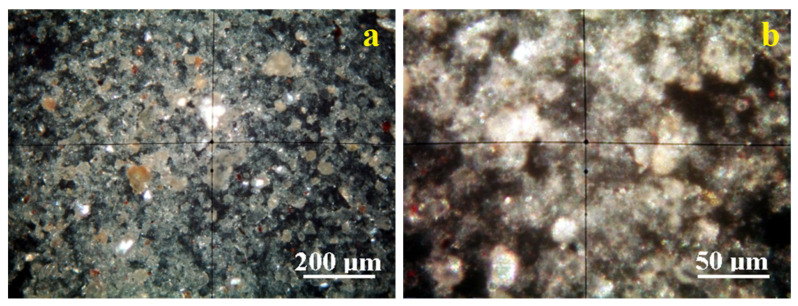
Mineralogical optical microscopy images observed in cross-polarized light for Pădurea Craiului clay catalyst: (**a**) microstructural overview; (**b**) microstructural detail.

The clay sample originating in the Pădurea Craiului area has a uniform microstructure with well-individualized small particles, Figure 6a. There occur a few quartz particles of about 20–30 µm having boulder-like shapes displaying their specific green–gray color and several bigger kaolinite particles of 5–10 µm having bright white appearance.

These bigger particles are surrounded by the finest fractions, which are better viewed in the microstructural detail displayed in Figure 6b. The kaolinite fine particles dominate the Pădurea Craiului clay. Their size ranges from 1 to 2.5 µm and has a bright white–bluish aspect. Some fine muscovite fractions occur with tabular particles colored in brown–pink shades and sizes in the range of 1–2.5 µm.

#### 2.1.5. Analysis of Clay Catalysts Nanostructure

**Figure 7:**
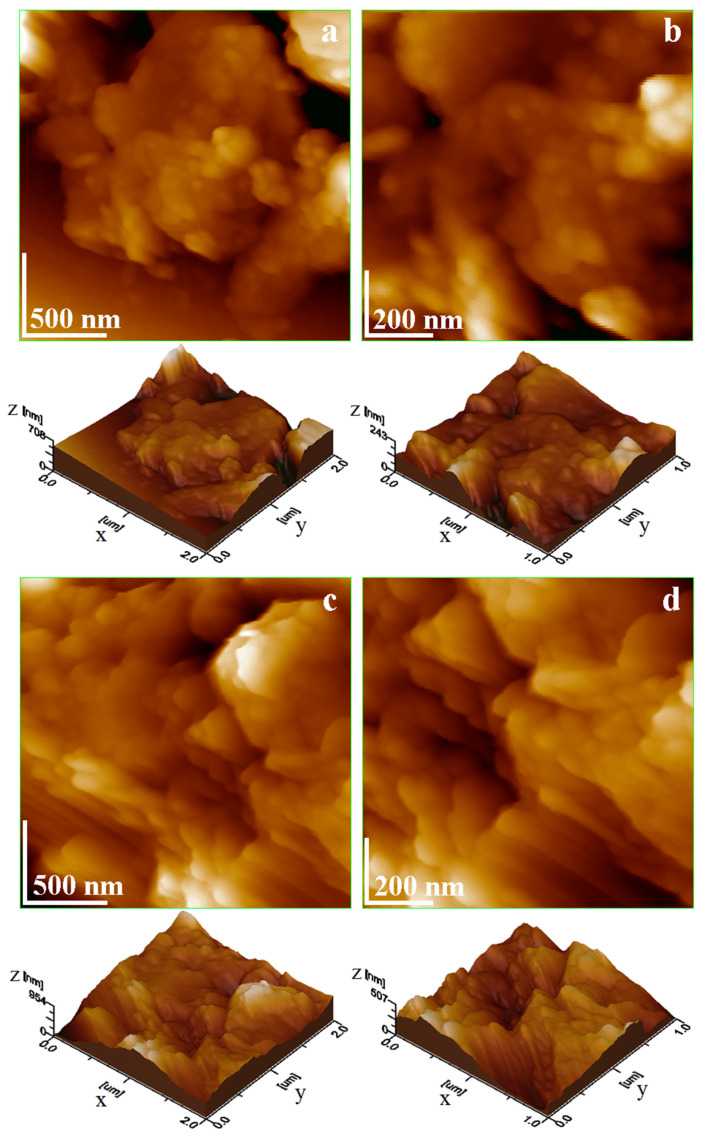
AFM topographic images of the nanostructured clay catalysts Vadu Crișului: (**a**) overall nanostructure; (**b**) nanoparticle details and Piatra Craiului; (**c**) overall nanostructure; (**d**) nanoparticles details. Three-dimensional profiles are presented below each topographic image.

Clay catalysts were properly dispersed into the reaction liquid oils. It penetrates the cluster’s pores and mobilizes fine fractions previously stacked into solid formations observed during SEM investigation, a fact allowing proper individualization of kaolinite fine particles. The agglomerated clusters are destructured, releasing well-individualized fine fractions in Brownian motion while traces of quartz particles are subjected to an almost instant sedimentation on the bottom of the vial. Glass slides were immersed vertically into the catalyst liquid dispersion, allowing particle adsorption. Thereafter, the adsorbed layers were naturally dried into a desiccator and further investigated by AFM, resulting in the topographic images in Figure 7.

The topography in Figure 7a is dominated by fine nanoparticles that are relatively uniform over the glass slide, forming a compact structure of kaolinite. Some large submicron muscovite platelets are observed on the upper corners of the scanned area. Therefore, the surface roughness is situated at 63.1 nm. The nanostructural detail in Figure 7b reveals the uniformly and compact adsorption film of kaolinite nanoparticles having sizes in the range of 40–60 nm. Their thickness could be appreciated by measuring the lateral size of obliquely oriented nanoparticles and is situated between 10 and 20 nm.

Piatra Craiului clay has a more irregular topography because of the kaolinite nanoparticles mixture with submicron muscovite platelets, Figure 7c. These are interlaced into a relatively random disposal as a consequence of free particle adsorption. However, the muscovite platelets are oriented relatively parallel to each other, and kaolinite nanoparticles are adsorbed over these arrangements. An AFM investigation effectuated by Avram and Birle 2024 reveals the ability of kaolinite nanoparticles to cover submicron features when exposed to liquid moisture [27], confirming the observed behavior in our sample. In fact, it affects the surface roughness, which is situated around 93.8 nm. The nanostructural details in Figure 7d show that Piatra Craiului kaolinite nanoparticles range from 60 to 80 nm, and their thickness is merely situated beside 20 nm. The size of submicron muscovite platelets ranges from 200 to 600 nm with a thickness of about 40 nm.

### 2.2. Yield of Thermal Degradation Products

Thermo-catalytic degradation products, abbreviated by type of plastic wastes, type of catalyst, and thermal degradation temperature, are presented in Table 2.

The experiments were conducted at 380 °C and 420 °C, and the obtained thermal degradation products were classified into three groups: liquid, gas, and residue [11]. As is known, the thermal degradation yield depends on many parameters such as experimental reactors, temperature, type of polymer, and catalyst type [29,30,31]. Temperature plays a major role in the distribution of the thermal degradation products obtained.

The product yields were calculated using Equations (1)–(3). The liquid fraction and the residue were weighed directly.

(1) $$L\left(wt.\%\right)=\frac{m_{L}}{M}\cdot 100$$

(2) $$R\left(wt.\%\right)=\frac{m_{R}}{M}\cdot 100$$

(3) $$G\left(wt.\%\right)=100-\left(L+R\right)$$

where $m_{L}$—the weight of liquid fraction, $m_{R}$—the weight of residue, *M*—the weight of plastic.

**Figure 8:**
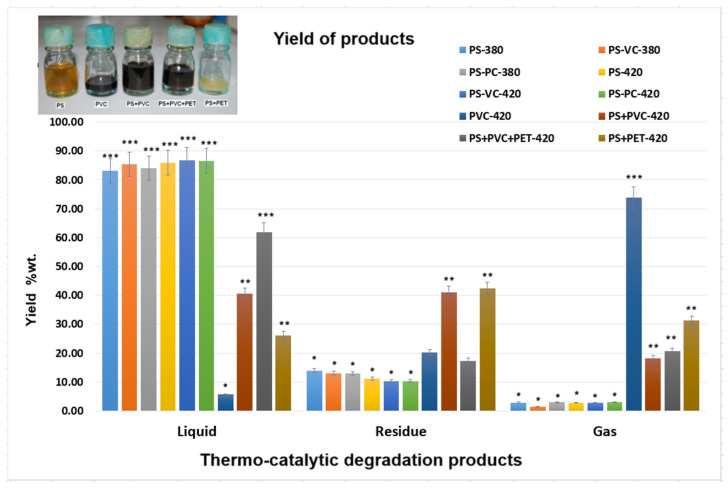
The yields of thermo-catalytic degradation products at 380 °C and 420 °C of waste plastics and, as detailed above, the color of some obtained liquid oils. Three different statistical groups: * group with reduced yield *p* < 0.05; ** group with average yield *p* < 0.05; *** group with increased yield *p* < 0.05; significant differences are observed between the three statistical groups at *p* < 0.05.

Thus, the yields obtained for the thermal degradation of plastic wastes are presented in Figure 8.

It can be observed that the colors of liquid oils depend on the type of plastic waste decomposed. The presence of vinyl polychloride is indicated by a dark color in the oils. The temperature and the type of catalyst influenced the colors of pyrolytic liquid oils [16].

The liquid yield increases with the temperature increase and in the presence of clay catalyst for degradation of PS waste. In the case of thermal degradation at 420 °C of PVC, it was obtained a very small yield of the liquid fraction (5.70, wt.%) and a large yield of gas fraction (73.95, wt.%) due to the presence of chlorine that was removed in the reaction together with other non-condensable gases. By comparison, the highest chlorine content (47.0 ± 1.4%) was eliminated at the decomposition of PVC in supercritical water at 400 °C [32].

Also, by thermal degradation of the mixture of plastic waste, PS+PET at 420 °C was obtained at just 26.18 wt.% of liquid fraction yield, and it is an important yield of residue of 42.52 wt.%. This temperature of 420 °C was relatively low for thermal degradation of PET [33].

In the case of the thermal degradation at 420 °C of a mixture of plastic wastes, the fractions yield of 62.00 wt.% of liquid, 17.36 wt.% solid and 20.63 wt.% gases was obtained from (PS+PVC+PET). Some authors obtained similar yields at thermal degradation at 450 °C of PS and PET plastic wastes [33].

The statistical analysis of the yield variation of thermo-catalytic degradation products showed that the PS yields had significant differences from the yields of the other plastics studied (*p* < 0.05). The types of plastic wastes have a great influence on their obtained liquid fraction yields.

### 2.3. GC × GC-qMS Analysis

Comprehensive two-dimensional gas chromatography is a very complex technique for the separation and identification of complex mixtures. In this study, a high-speed scan single quadrupole mass spectrometry was used to identify individual components in an ordered manner according to their volatility and polarity in liquid oils obtained by the thermal degradation of plastic wastes. The compounds were identified by comparison of the mass spectrum to the NIST98 MS library spectra. Assigning spots was carried out either by spectrum matching-based searches with the mentioned databases and similarity score (the probabilities that exceeded 50%) considering the RT 1st D (min)—Retention time of first dimension (min) and RT 2nd D (s)—Retention time of second dimension (s).

The area (%) of identified compounds from liquid oils of PS waste and from liquid oil of plastic waste mixture were compared.

**Figure 9:**
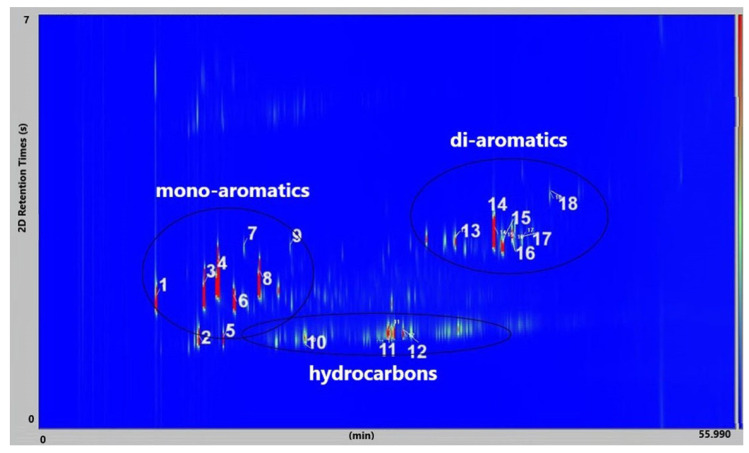
GC × GC-qMS color plot of liquid oil obtained by thermal degradation of PS waste in presence of Vadu Crișului clay catalyst at 420 °C.

Figure 9 presents the GC × GC-qMS chromatogram obtained by thermal degradation of PS waste in the presence of Vadu Crișului clay catalyst at 420 °C. Thus, all samples of thermal and catalytic degradation of PS waste were identified into eighteen compounds and classified into three groups: hydrocarbon (linear), mono-aromatics, and di-aromatics. You can see this in Figure 9 and Table 3.

**Figure 10:**
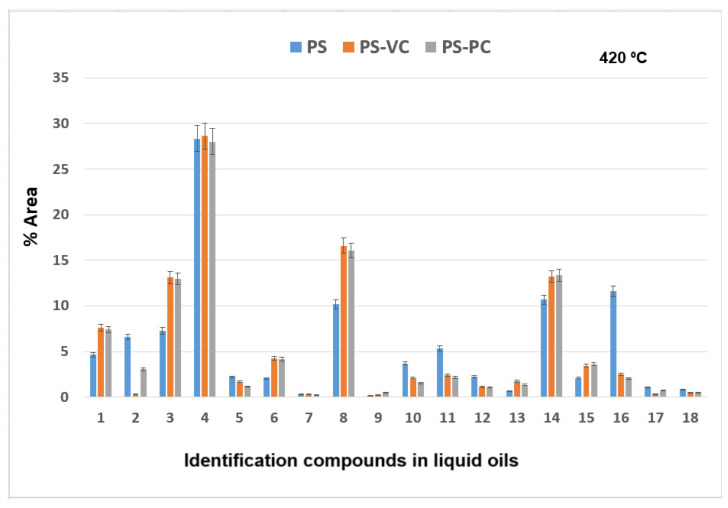
The identified compounds (% Area) in the liquid oils obtained by thermo-catalytic degradation of PS waste at 420 °C.

The results of GC × GC-qMS analysis of identified compounds (% Area) of the liquid oils obtained by thermal and catalytic degradation of PS waste at 420 °C are shown in Figure 10.

Major compounds were obtained: styrene (compound **4**) in the range of 28.01–28.61%, α-methyl styrene (compound **8**) in the range of 10.19–16.59% area, and benzene, 1,1′-(1,3-propanediyl) bis-(compound **14**) in range of 10.67–13.39%. The 11.61% of benzene,1,1′-(2-butene-1,4-dyil) bis (compound **16**) was obtained at thermal degradation of PS at 420 °C.

**Figure 11:**
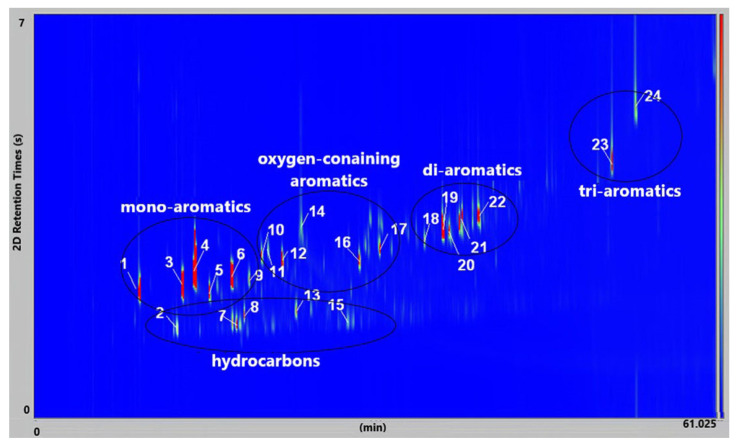
GC × GC-qMS color plot of liquid oil obtained from thermal degradation at 420 °C of mixture of plastic waste (PS+PVC+PET).

Figure 11 presents the GC × GC-qMS chromatogram of liquid oil obtained by thermal degradation of a mixture of plastic waste (PS+PVC+PET) at 420 °C. Twenty-four compounds were identified and shown in Table 3.

The identified compounds (Figure 11, Table 4) were presented in five groups: hydrocarbons (linear), mono-aromatics, oxygen-containing aromatic, di-aromatics, and tri-aromatics. The exception is compound no. 12, which is a chloro-containing aromatic compound.

At a thermal degradation at 420 °C of the mixture of plastic waste (PS+PVC+PET), twenty-four compounds that are specific to the type of plastic waste that was decomposed were identified.

In Table 3, the identified compounds were those specific to the degradation of PS (compounds **1**, **3**, **4**, **6**), PVC (compounds **7**, **8**, **12**, **15**, **19**–**24**), and PET (compounds **10**, **11**, **14**, **16**, **17**) given that the mass ratio of the mixture of plastic waste of PS: PVC: PET = 18:3:4, wt/wt, was used for thermal degradation at 420 °C.

Toluene, ethylbenzene, styrene, and α-methyl styrene, obtained from the decomposition of PS waste, were found as the majority in these liquid oils because the PS was present as principal waste. Then, the other compounds identified, as well as benzene, 4-(chloromethyl)-1,2-dimethyl-, benzoic acid, benzene, 1,1′-(1,3-propanediyl) bis-, benzene,1,1′-(2-butene-1,4-diyl) bis, 1-propene,3-(2-cyclopentenyl)-2-methyl-1,1-diphenyl, were obtained at the decomposition of PVC and PET waste.

## 3. Discussion

In this study, the natural clay catalysts used for the thermal degradation of PS waste are the kaolinite type. The Vadu Crișului clay catalyst was partially characterized in our previous work with good results [5]. The presence of a catalyst at the thermal degradation of PS waste determines the increase in the yield of the liquid fraction. Some authors used the Fe- restructured natural clay for thermo-catalytic conversion of a mixture of PE, PP, PS, PVC, and PET plastics, with the highest liquid yields [32,33,34].

The characterization of these clay catalysts by nitrogen adsorption–desorption isotherms shows an isotherm predominantly type I (Langmuir) combined with type IV isotherm, according to the International Union of Pure and Applied Chemistry (IUPAC) classification [35]. This indicates that the adsorption mechanism transforms from the monomolecular layer adsorption to multilayer adsorption because there is no restriction of adsorption space on the surface of the adsorbent. The adsorption isotherm rises slowly in low relative pressure and then bulges while the slope of the isotherms declines. When the relative pressure increases to a special turning point, the isotherm nearly becomes a straight line, and the slope becomes a constant [36]. The adsorption isotherms suggest that the catalysts are micro- and mesoporous materials. The microporous nature of these catalysts is indicated by the low adsorption volume at low relative pressure (P/Po < 0.1). When the relative pressure is increased, capillary condensation takes place in mesopores, showing type IV isotherm behavior with its characteristic H4 hysteresis loop. This type of hysteresis loop is often associated with narrow slit-like pores that are usually found in solids consisting of aggregates or agglomerates of particles. Additionally, starting with the high relative pressures P/Po > 0.8, a rapid growth of the N_2_ amount adsorbed can be observed, which indicates a secondary porosity (inter-particles) associated with meso- and macropores existence [35,36,37].

Clay catalysts were selected among possible environmentally friendly, cheap, and common catalysts in the thermo-catalytic conversion of polystyrene/plastic waste. The moderate acidity and the presence of both Lewis and Brønsted acid sites on the surface of clays favor heavier hydrocarbons in liquid products of reactions occurring in their pores [35,37].

The fine microstructure proven by the POM microscopy evidences the finest fractions of kaolinite in both clays originating in Vadu Crisului and Pădurea Craiului. These are obviously sported by their specific interference color, ranging from bright white to pale blue depending on the particle’s orientation regarding the microscope’s optical axis [29,38,39].

Quartz interference colors are green–gray, and muscovite has brown–pink shades [38,39]. Quantitative measurements performed on the microstructural details in Figure 5b and Figure 6b allow for establishing the mineral amounts. Thus, Vadu Crișului has about 91% kaolinite, 6% muscovite, and 3% quartz. Pădurea Craiului exhibits a wide particulate heterogeneity induced by fewer larger quartz and kaolinite particles. Therefore, it is mandatory to focus on the microstructural detail revealing the finest particle distribution, which indicates a composition of 82% kaolinite, 11% muscovite, and 7% quartz. Kaolinite and muscovite have tabular habits of the crystals with parallel sheets, as observed in SEM images, Figure 3. Thus, the fine microstructural clusters formed by the kaolinite and muscovite particles exhibit a large reactive surface able to interact with the oil moisture. Quartz particles did not have catalytic influence due to their inert weights. Their occurrence might influence the catalyst yield.

The variable amount of quartz in our local clay catalysts represents a limitation of the present research, which makes it dependent on the local sources. Clay soils similar to ours are found almost anywhere on the earth; therefore, the present study has great importance for researchers in the field because it highlights the importance of these cost-effective sources of local availability all over the globe.

All these characteristics of clay catalysts as well as the thermal degradation temperature, influenced the yield of liquid oils of degradation of PS.

Temperature plays a major role in the distribution of thermal degradation products obtained [32,40]. Literature data [16,32,34,40] indicates a minimum temperature of 350 °C to sustain the thermo-catalytic degradation process of plastic wastes. Therefore, we opt for a close temperature range of (380 °C/420 °C) to ensure an optimal yield of the degradation process along with a low energy consumption. Thus, in this paper, it can be observed that in the case of thermo-catalytic degradation of PS, the yields of obtained liquid fractions showed high values between 83.19 wt.% for PS-380 and 86.85 wt.% for PS-VC-420 samples. Also, the residue fractions of thermo-catalytic degradation of PS decrease with the increase of the degradation temperature. Similar results were obtained and presented in our previous papers by using different types of catalysts [5].

In literature, a high yield of oil of 71.0% was obtained by using montmorillonite as clay catalyst in the pyrolysis of mixed plastic [14]. And the Olivine and CaO catalysts used for catalytic pyrolysis of PS were capable of increasing the pyrolytic oil up to 74.5 and 71.4 wt.%, respectively [16]. Thus, with the increase in the degradation temperature of plastic waste, the liquid fraction yields are increased due to more condensable compounds forming [33,40].

GC × GC-MS characterization of petroleum products, pyrolytic oils from plastic waste, or complex olefin mixtures has been studied in the literature [3,19,23]. In the pyrolysis oil in terms of both group type and carbon number were found: hydrocarbon groups (*n*-paraffins, *iso*-paraffins, olefins and naphthenes, monoaromatics, naphtheno-aromatics, diaromatics, naphthenodiaromatics, triaromatics, naphtheno tri-aromatics and tetra-aromatics), nitrogen (nitriles, pyridines, quinolines, indole, caprolactam, etc.), sulfur (thiols/sulfides, thiophenes/disulfides, benzothiophenes, dibenzothiophenes, etc.) and oxygen-containing compounds (ketones, phenols, aldehydes, ethers, etc.) [3,21].

In our study, the liquid oil characterization of PS waste shows the presence of eighteen identified compounds (Figure 9 and Table 3). Also, in the liquid oil of a mixture of plastic waste (PS+PVC+PET) at 420 °C, were identified five groups of compounds (hydrocarbons (linear), mono-aromatics, oxygen-containing aromatic, di-aromatics and tri-aromatics) (Figure 11, Table 4).

Analysis of mixed plastic pyrolysis oil by comprehensive two-dimensional gas chromatography coupled with low- and high-resolution time-of-flight mass spectrometry showed chemical class separation according to the carbon group C7–C39 [18]. The complete group-type to component-by-component analysis of fossil and alternative fuels of different synthetic and fossil fuels and their crude products was achieved by GC × GC-MS [23].

The results reveal that the group-type separation better highlights the compounds obtained depending on the type of decomposed polymer. Many other compounds were separated in pyrolytic liquid oils but could not be identified due to technical limitations. The GC × GC-qMS method was an appropriate solution for the evaluation of these liquid oils obtained at thermal and catalytic degradation of waste plastics.

The information obtained from this study provides a new insight into the composition of liquid oils obtained from the thermal and catalytic degradation of plastic waste in order to reconvert them in the styrene monomer or the other chemicals and considerably reduce the polymer wastes from environmental factors. The natural clay catalysts increase the environmentally friendly aspect of the recovering technological process.

## 4. Materials and Methods

### 4.1. Materials

The plastic waste of PS, PET, and PVC were obtained from vessel detergent boxes and disposable glasses and plates, beverage bottles, pipes, and other PVC products manufactured by Romanian companies. These plastic wastes were cut into small pieces, approximately 5 × 5 mm, and used individually and in the mixture. The proportions used by us in the mixture of plastic waste were based on the composition proposed by other authors as representative of municipal plastic wastes in Europe [24].

Two kaolinite powders exploited in the Transylvania area (Romania): Vadu Crișului (VC) and Pădurea Craiului (PC) were used as catalysts for the degradation of PS at a concentration of 10% in mass (catalyst/PS). The catalysts were subjected to a heating treatment in an oven in two stages. The first was made at 200 °C for 2 h to remove the adsorbed water from the mesopores, then the second heat treatment at 500 °C for 5 h to activate the active centers followed. Then, the catalysts were kept in a desiccator for the experiments.

### 4.2. Physicochemical Investigation Methods of Catalysts

The surface area and porosity characteristics of the calcined catalysts were determined by nitrogen adsorption–desorption isotherms at 77 K using a Micromeritics TriStar II 3020 instrument (TriStar Technologies, El Segundo, CA, USA). The adsorption desorption curves of the isotherms were recorded, and the surface area measurements were performed according to the BET (Brunauer–Emmett–Teller) method. The pore size distributions were obtained applying the BJH (Barrett, Joyner, Halenda) method on the isotherm desorption branch, and the micropores area and volume were obtained applying the t-plot (de Boer) method.

External surface, morphology, and structure of catalysts were visualized by Scanning Electron Microscopy (SEM), and the chemical compositions were determined by the Energy Dispersive X-Ray Analysis (EDX) method using a QUANTA 133 Electron Microscope (FEI Company, Hillsboro, OR, USA).

Structural information of catalysts was obtained by Fourier Transform Infrared Spectroscopy (FTIR) using a JASCO-610 spectrophotometer produced by JASCO International Co., Ltd., Tokyo, Japan. Each spectrum was registered in the 4000–400 cm^−1^ wave number range, using the KBr pellet technique, in order to evidence the specific absorption bands correlated to the chemical compounds.

Mineral particles distribution within the catalyst powder was assessed through Polarized Optical Microscopy (POM). The images were obtained using a Laboval 2 Microscope produced by Carl Zeiss, Oberkochen, Germany, under the cross-polarized light inspection of the samples. The images were acquired with a digital camera of 10 megapixels produced by Samsung, Seoul, Republic of Korea.

Clay catalyst nanostructure was investigated through Atomic Force Microscopy (AFM) on a JSPM 4210 Scanning Probe Microscope (Jeol, Tokyo, Japan) operated in tapping mode with NSC 15 Hard cantilever (MikroMasch, Tallinn, Estonia) with resonant frequency of 325 kHz and force constant of 40 N/m. The obtained topographical images were analyzed with WinSPM 2.0 software (Jeol, Tokyo, Japan), and surface roughness and nanoparticle size were measured.

### 4.3. Thermal Degradation Procedure

Thermal degradation was performed using the procedure described in our previous work. Thermal degradation of the plastic waste was performed in a tubular glass reactor (175 mm × 33 mm ID) heated externally by an electric furnace with a temperature controller. The plastic waste was added to the reactor. In the catalytic degradation process, a mass ratio of 10:1, wt/wt, of plastic waste and catalyst were used. Before the starting of the experiments, the nitrogen gas was continuously passed through the installation with a flow rate of 30 mL·min^−1^ for 10 min to remove the air. Then it was followed the thermal decomposition step by heated at a rate of 10 °C·min^−1^ up to the desired temperature. The formed gaseous products were passed through a water-cooled condenser (0–4 °C), and the condensable gases were collected as a liquid product. The incondensable gases were collected in a special bag as a gaseous product. The small quantity of residue and the used catalyst remain in the glass reactor. The thermal degradation procedure was carried out at temperatures of 380 °C and 420 °C, respectively. The yields of obtained products were calculated. The liquid fractions were subjected to dehydration with MgSO_4_ in order to remove any traces of water that could affect the gas chromatographic analysis. All datasets were analyzed using the one-way ANOVA test (α = 0.05), performed with Origin2019b Graphing and Analysis software (OriginLab, Northampton, MA, USA).

### 4.4. Bidimensional Gas Chromatography Analysis of Liquid Fractions

The GC × GC-qMS analysis was performed with TRACE GC × GC comprehensive two-dimensional gas chromatograph (Thermo-Electron Corporation, Courtaboeuf, France). The instrument was equipped with a cryogenic dual-stage CO_2_ jet modulator and coupled to an ISQ mass selective detector, an autosampler TriPlus model, and HyperChrom Software version 1.1. For GC × GC-qMS analysis, helium of high purity at a constant flow rate of 1 mL·min^−1^ was used as carrier gas. For mass spectrometry, the frequency of acquisition was 50 Hz, and for data collection, a total ion current (TIC) MS signal was scanned between 40 and 350 *m*/*z*. The MS transfer line temperature was 280 °C, and the ion source temperature was 200 °C. The MS ionization mode was electron ionization using a voltage of 70 eV. The first column was a non-polar capillary column Factor Four VF1-MS, 15 m × 0.25 mm I.D. × 0.25 µm film thickness (100% dimethylpolysiloxane) purchased from Varian (Restek Lisses, France). The second column was a moderate polar column DB-17, 1.5 m × 0.10 mm ID × 0.1 μm (film thickness), (50% phenyl − 50% methylpolysiloxane) purchased from Agilent Technologies (Les Ulis Cedex, France). The acquisition of data was performed using the X-Calibur software 4.3, and the GC × GC representation was realized using the Chrom-Card software version 2.3 (ThermoElectron Corporation, Courtaboeuf, France).

The analyses were performed with a gradient temperature program. The oven temperature was programmed starting at 40 °C·min^−1^ (held 10 min) and increased from 5 °C·min^−1^ to 260 °C (held 5 min). The injector temperature and the detector temperature were 260 °C and 280 °C, respectively. The sample (1 µL) was injected in a split ratio of 1:100 into the SSL injector. Modulation period was 7 s. Compounds were identified by comparison of the mass spectrum to the NIST98 MS library spectra version 1998 (National Institute of Standard and Technology, Gaithersburg, MD, USA).

## 5. Conclusions

In this study, the yield fraction obtained by the thermal and catalytic degradation of some waste plastics (polystyrene, polyvinyl chloride, polyethylene terephthalate), individually or in a mixture, or in the presence of natural clay catalysts, was evaluated. The natural clay catalysts from the Transylvania area are the kaolinite type and were well characterized, as evidenced by the micro- and mesopores.

The liquid yields obtained by thermal and catalytic degradation of PS waste have been calculated between 83.19 wt.% and 86.85 wt.%. Using the Romanian natural clay catalysts for the study of the thermal and catalytic degradation of PS waste could reduce catalytic process costs and obtain an important quantity of styrene monomer.

By thermal degradation of PVC waste at 420 °C, a very small yield of the liquid fraction of 5.70, wt.% was obtained because the highest content of chlorine gas was removed. Moreover, for the mixture of PS+PET waste, 26.18 wt.% liquid fraction yield was obtained, and for the mixture of PS+PVC+PET waste, 62.00 wt.% liquid fraction yield was obtained.

Identification of the compounds of the pyrolytic liquid oils by using GC × GC-qMS technique reveals the three or five groups of compounds—hydrocarbons (linear), mono-aromatics, oxygen-containing aromatic, di-aromatics and tri-aromatics—depending on the type of decomposed plastic waste.

The information obtained from this study provides new insight into the composition of the liquid oils obtained from the thermal and catalytic degradation of plastic waste for the purpose of reconverting them into styrene monomers or other chemicals or to be used in various technological applications for obtaining different types of copolymers: acrylonitrile butadiene styrene, styrene-acrylonitrile, acrylonitrile styrene acrylate, etc. These copolymers can be found in many areas, including automotive components, construction, telecoms, household appliances, and sports and leisure equipment.

## Figures and Tables

**Table 1:** Surface area and porosity characteristics of studied catalysts.

Clay Catalyst	^a^ Specific Surface Area, BET, [m^2^ g^−1^]	^b^ Micropore Area, [m^2^ g^−1^]	^b^ Micropore Volume, [cm^3^ g^−1^]	^c^ Total Pore Area, [m^2^ g^−1^]	^c^ Average Pore Diameter, [nm]	^c^ Total Pore Volume, [cm^3^ g^−1^]
Vadu Crișului	27.80	9.48	0.0013	28.32	15.30	0.108
Pădurea Craiului	21.01	7.15	0.0009	22.21	12.11	0.067

^a^ BET (Brunauer–Emmett–Teller) method; ^b^ t-plot (de Boer) method; ^c^ BJH (Barrett, Joyner, Halenda) desorption method.

**Table 2:** The abbreviation of studied samples obtained by thermo-catalytic degradation of plastic wastes.

Abbreviation	^1^ Thermo-Catalytic Degradation Products (Plastic Waste–Catalyst–Temperature)
PS-380	Polystyrene–380 °C
PS-VC-380	polystyrene–Vadu Crișului clay–380 °C
PS-PC-380	polystyrene–Pădurea Craiului clay–380 °C
PS-420	polystyrene–420 °C
PS-VC-420	polystyrene–Vadu Crișului clay–420 °C
PS-PC-420	polystyrene–Pădurea Craiului clay–420 °C
PVC-420	polyvinyl chloride–420 °C
PS+PVC-420	polystyrene + polyvinyl chloride–420 °C, (1:1, w/wt)
PS+PVC+PET-420	polystyrene + polyvinyl chloride + polyethylene terephthalate–420 °C, (18:3:4, wt/wt)
PS+PET-420	polystyrene + polyvinyl chloride + polyethylene terephthalate–420 °C, (1:1, wt/wt)

^1^ Thermo-catalytic degradation products—liquid oil, residue, gas, obtained by thermo-catalytic degradation of plastics waste.

**Table 3:** Compounds identified in liquid oils obtained by thermal degradation PS waste in presence of Vadu Crișului clay catalysts at 420 °C.

No. Crt.	Component Name	Molecular Structure	* RT 1st D (min)	** RT 2nd D (s)	PS-VC-420 % Area ± SD
1	Toluene/C_7_H_18_	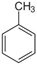	9.45	2.30	7.61 ± 0.15
2	2,4-Dimethyl-1-heptene/C_9_H_18_	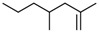	12.83	1.70	0.32 ± 0.01
3	Ethylbenzene C_8_H_10_	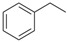	13.30	2.41	13.10 ± 0.34
4	Styrene/C_8_H_8_	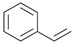	14.35	2.93	28.11 ± 1.65
5	Isobutyl Cyclohexane/C_10_H_20_	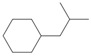	14.82	1.63	1.66 ± 0.04
6	Benzene, (1-methylethyl)-/C_9_H_12_	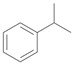	15.63	2.22	4.25 ± 0.07
7	Benzaldehyde/C_7_H_6_O	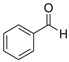	16.45	3.15	0.30 ± 0.01
8	A-Methyl styrene/C_9_H_10_	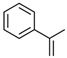	17.73	2.63	16.59 ± 0.69
9	Benzene, ethenylmethyl-/C_9_H_10_	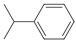	20.18	3.19	0.25 ± 0.02
10	Nonane, 5-methyl-/C_10_H_22_	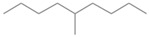	21.35	1.67	2.11 ± 0.11
11	1-Undecene-7-methyl/C_12_H_24_	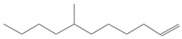	28.12	1.78	2.41 ± 0.32
12	2-Methyldodecane/C_13_H_28_	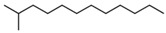	29.28	1.74	1.12 ± 0.02
13	2,6-Diethylnaphthalene/C_14_H_16_	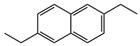	33.48	3.22	1.74 ± 0.02
14	Benzene, 1,1′-(1,3-propanediyl) bis-/C_15_H_16_	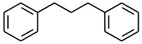	36.63	3.44	13.22 ± 1.01
15	Benzene, 1,1′-(1-methyl-1,3-propanediyl) bis-/C_16_H_18_	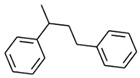	37.33	3.15	3.42 ± 0.91
16	Benzene,1,1′-(2-butene-1,4-dyil) bis/C_16_H_16_	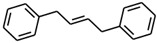	38.03	3.59	2.46 ± 0.44
17	Butene,1,1′(3-methyl-1-propene-1,3diyl) bis/C_16_H_16_	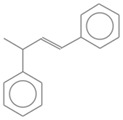	38.92	3.3	0.36 ± 0.21
18	Benzene,1,1′-(1,1,2,2-tetramethyl-1,2-ethanediyl) bis	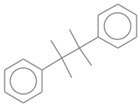	41.39	4.11	0.41 ± 0.03

* RT 1st D (min)—Retention time of first dimension (min); ** RT 2nd D (s)—Retention time of second dimension (s); SD—Standard deviation of three analyses by GC × GC-MS.

**Table 4:** Compounds identified in liquid oils obtained by thermal degradation at 420 °C of mixture of plastic waste (PS+PVC+PET).

No. Crt.	Component Name	Molecular Structure	* RT 1st D (min)	** RT 2nd D (s)	PS+PVC+PET, (Area %) ± SD
1	Toluene/C_7_H_8_	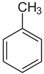	9.45	2.30	5.30 ± 0.35
2	2,4-Dimethyl-1-heptene/C_9_H_18_	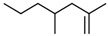	12.83	1.59	1.30 ± 0.05
3	Ethylbenzene/C_8_H_10_	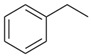	13.30	2.37	9.16 ± 1.65
4	Styrene/C_8_H_8_	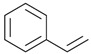	14.35	2.96	29.49 ± 3.85
5	Benzene, (1-methylehyl)-/C_9_H_12_	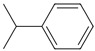	15.63	2.22	1.91 ± 0.09
6	α-Methyl styrene/C_9_H_10_	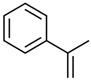	17.73	2.52	8.35 ± 1.38
7	*cis* 3-Decene/C_10_H_20_	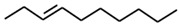	18.08	1.67	1.41 ± 1.25
8	1-Undecene, 5-methyl/C_12_H_24_	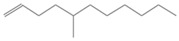	18.78	1.85	1.23 ± 0.82
9	Benzene, ethenylmethyl-/C_9_H_10_	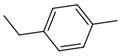	19.25	2.44	1.19 ± 1.12
10	Acetophenone/C_8_H_8_O	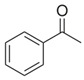	20.30	2.85	1.43 ± 1.33
11	α-Ethyl-α-methylbenzyl alcohol/C_10_H_14_O	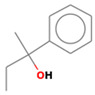	20.88	2.96	0.89 ± 0.69
12	Benzene, 4-(chloromethyl)-1,2-dimethyl-/C_9_H_11_Cl	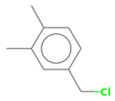	22.17	2.81	2.74 ± 1.74
13	Dimethyl octanol/C_10_H_22_O	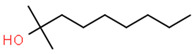	23.45	1.93	1.47 ± 0.63
14	Benzoic acid/C_7_H_6_O_2_	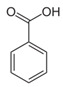	23.92	3.41	3.73 ± 1.77
15	1-Undecene, 7-methyl/C_12_H_24_	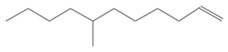	28.00	1.74	1.26 ± 1.65
16	2,4,6-Trimethyl-4-phenyl-1,3-dioxane/C_10_H_10_O	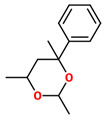	29.05	2.81	1.96 ± 2.01
17	2,5,5-Trimethyl-2-phenyl-1,3-dioxane/C_13_H_18_O_2_	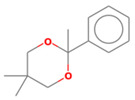	30.92	3.00	1.71 ± 1.88
18	Diphenyletane/C_14_H_14_	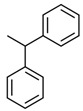	34.88	3.11	1.05 ± 0.65
19	Benzene, 1,1′-(1,3-propanediyl) bis-/C_15_H_16_	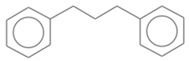	36.52	3.37	6.09 ± 2.21
20	Benzene, 1,1′-(1-methyl-1,3-propanediyl) bis-/C_16_H_18_	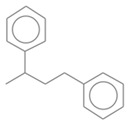	37.10	3.30	2.33 ± 1.64
21	Benzene,1,1′-(2-butene-1,4-dyil) bis/C_16_H_16_	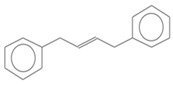	38.03	3.44	2.95 ± 2.01
22	Benzene,1,1′-3-methyl-1-propene-1,3-diyl) bis-/C_16_H_16_	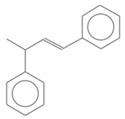	39.78	3.56	4.27 ± 1.98
23	(2,3-Diphenylcyclopropyl) methyl phenyl sulfoxide, trans-/C_22_H_20_S	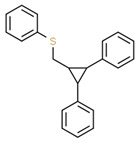	51.68	4.48	4.08 ± 1.17
24	1-Propene, 3-(2-cyclopentenyl)-2-methyl-1,1-diphenyl/C_21_H_22_	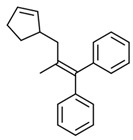	53.78	5.41	4.70 ± 2.56

* RT 1st D (min)—Retention time of first dimension (min); ** RT 2nd D (s)—Retention time of second dimension (s); SD—Standard deviation of three analyses by GC × GC-MS.

## Data Availability

The original contributions presented in this study are included in the article. Further inquiries can be directed to the corresponding author(s).

## Abbreviations

The following abbreviations are used in this manuscript:

PS	polystyrene waste
PVC	polyvinyl chloride waste
PET	polyethylene terephthalate
SEM	Scanning electron microscopy
FTIR	Fourier transform infrared spectrometry
POM	Polarized optical microscopy
AFM	Atomic force microscopy
GC × GC-qMS	Comprehensive two-dimensional gas chromatography coupled with single quadrupole mass spectrometry
